# Combined Central and Peripheral Demyelinating Disease With Good Response to B-Cell Depleting Therapy

**DOI:** 10.7759/cureus.14690

**Published:** 2021-04-26

**Authors:** Seraj Makkawi, Faisal Yonbawi, Yousef Qari, Morad Aljinaid

**Affiliations:** 1 College of Medicine, King Saud Bin Abdulaziz University for Health Sciences, Jeddah, SAU; 2 Medicine, King Abdullah International Medical Research Center, Jeddah, SAU; 3 Department of Medicine, Ministry of the National Guard-Health Affairs, Jeddah, SAU

**Keywords:** combined central and peripheral demyelination, rituximab, demyelination, multiple sclerosis, chronic inflammatory demyelinating polyradiculoneuropathies, neurology, saudi arabia

## Abstract

Combined central and peripheral demyelination (CCPD) is a rare disorder characterized by demyelinating lesions in both the central and peripheral nervous systems. The following case report is of a 29-year-old man who presented with a three-month history of progressive lower and upper limb weakness associated with facial and arm tremor, as well as urinary hesitancy. Brain and spine magnetic resonance imaging showed multiple demyelinating plaques. Nerve conduction studies revealed evidence of demyelination with severe prolongation of distal motor latencies and reduced conduction velocities. The patient received plasmapheresis and high-dose corticosteroids, which lead to clinical improvement. A rituximab infusion protocol was subsequently started, and the patient received two cycles. There was a significant functional improvement upon the use of rituximab. This study reports a rare neurological disease entity and highlights the necessity for conducting larger studies to optimally demonstrate the efficacy of rituximab in CCPD.

## Introduction

Combined central and peripheral demyelination (CCPD) is a rare disorder, and our current knowledge is based on data derived from case reports or small case series [1].

Multiple sclerosis (MS) is a chronic autoimmune disease that is confined to the central nervous system (CNS) [2]. The disease course is characterized by inflammation, demyelination of the CNS, gliosis, and eventually, neuronal loss. Furthermore, degeneration of distal oligodendrocytes processes and their apoptosis are part of MS pathophysiology. MS can cause a vast range of neurological symptoms based on the unique site of the lesion. It has a multifactorial etiology and a prevalence of 2.5 million individuals all over the globe.

On the other hand, chronic inflammatory demyelinating polyradiculoneuropathy (CIDP) is an acquired immune-mediated disorder that typically targets the peripheral nervous system (PNS) [3]. Myelin components of the PNS, which are generated by Schwann cells, are attacked by T-cell-mediated actions and humoral immune mechanisms, which underlie the etiology of this disease. CIDP usually develops over more than eight weeks with a diverse range of clinical presentations.

In general, CCPD involves disparate conditions with acute, relapsing, and chronic subtypes [4]. This raises a concern of either addressing this disease as a separate entity due to a shared immunopathogenic mechanism, or a potential coincidence between two unrelated demyelinating disorders, MS and CIDP.

In this article, we present a case of CCPD disease with a good response to B-cell depleting therapy.

## Case presentation

A 29-year-old man presented with a three-month history of progressive lower-limb weakness and numbness extended to involve upper limbs one month before the presentation. Weakness was more prominent distally, and more on the left than right. It was associated with facial and arm tremors and urinary hesitancy. The patient’s symptoms had a fluctuating course with no particular triggers. There was no history of viral infection before symptom onset. The patient had no history of other neurological disorders. The patient had no history of fever, weight loss, or night sweats, and the systemic review was unremarkable.

On physical examination, the patient was vitally stable. There was a relative afferent pupillary defect on the left eye with a reduced visual acuity of 20/25 on the same eye. Otherwise, visual field, fundoscopy, and other cranial nerves examinations were normal. The patient had a reduced muscle strength graded as 4 on hand gripping, 4 on wrist flexion, 4 on wrist extension, 4 on elbow flexion, 3 on elbow extension, 4 on hip flexion and extension, 4 on knee flexion and extension, and bilateral foot drop. Deep tendon reflexes were all absent. There was a stocking distribution of reduced sensation, particularly to vibration and proprioception. The patient had an Expanded Disability Status Scale (EDSS) score of 6.

**Figure 1:**
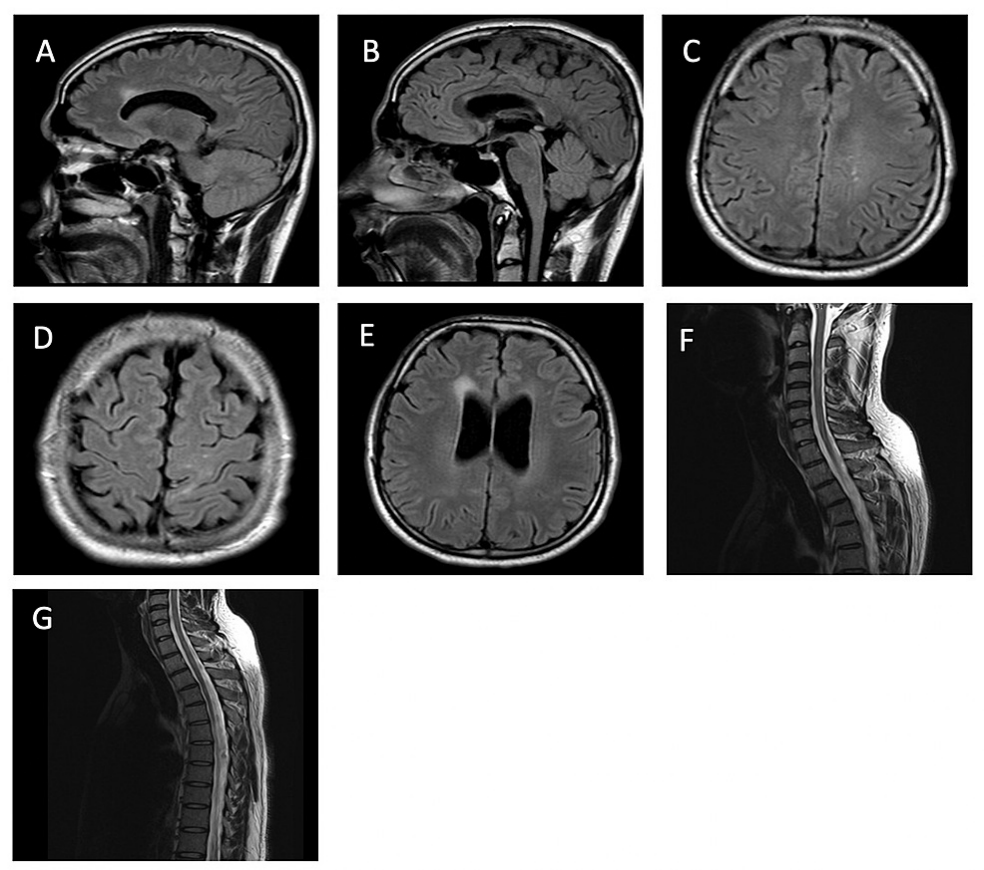
MRI brain and spinal cord. (A-B): Sagittal view of brain MRI (FLAIR sequence) showing multiple hyperintense signal changes in the corpus callosum. (C-E): Axial view of the brain MRI (FLAIR sequence) showing left cortical (parietal), left subcortical, and deep periventricular hyperintense signal changes, respectively. (F-G): Sagittal view of spine MRI showing multilevel eccentric signal changes of the spinal cord. MRI: magnetic resonance imaging; FLAIR: fluid-attenuated inversion recovery

Brain magnetic resonance imaging (MRI) showed multiple demyelinating plaques involving the corpus callosum, right frontal lobe, deep periventricular white matter, and external capsule (Figures 1A-E). Cervical and thoracic spine MRI showed multilevel eccentric signal alterations without any enhancement (Figures 1F-G).

Nerve conduction studies detected demyelinating polyneuropathy with severe prolongation of distal motor latencies and reduced conduction velocities over the bilateral median, left ulnar, and bilateral tibial nerves (Table 1). Electromyography showed neurogenic motor units with minimal denervation potentials. Visual evoked potentials revealed borderline prolongation of p100 wave latency, left more than right.

**Table 1:** Nerve conduction studies of the motor nerves.

Nerve	Site		Latency (ms)	Amplitude (mV)	Conduction velocity (m/s)
Median	Right	Wrist	17.2	1.9	
		Elbow	25.8	1.5	27.9
	Left	Wrist	27.2	1.5	
		Elbow	40.8	1.2	18.4
Ulnar	Left	Wrist	15.4	2.2	
		Elbow	22.8	1.8	31.1
		Above Elbow	25.4	1.7	38.5
Peroneal	Left	Absent			
Tibial	Right	Ankle	29.0	0.423	
		Popliteal	41.6	0.316	31.7
	Left	Ankle	24.4	0.567	
		Popliteal	37.2	0.441	29.7

Cerebrospinal fluid (CSF) analysis revealed absent white count, elevated protein level (1.7 g/L), elevated albumin level (584 mg/L), elevated IgG level (78.9 mg/L), elevated CSF-to-serum albumin ratio (14.5), with negative CSF and blood oligoclonal bands. Serum albumin and serum IgG levels were normal. CSF cytology was negative for malignant cells. CSF angiotensin-converting enzyme level was normal. Anti-neurofascin IgG (NF155 and NF186), aquaporin-4, and anti-MOG antibodies were negative. Extensive investigations for other potential causes were negative, including serology for human immunodeficiency virus, hepatitis B and C, cytomegalovirus, vasculitis, and paraneoplastic workup (anti-Hu, anti-Ri, anti-Yo, and anti-CV2/RMP5, DPPX, CASPR2, LGI1 antibodies). Computed tomography (CT) scans of the chest, abdomen, and pelvis were normal.

Upon hospital admission, the patient showed clinical improvement in the weakness of his upper and lower limbs after receiving plasmapheresis. This was followed by a course of prednisolone for four months. However, the patient’s symptoms deteriorated with the steroid taper as he developed a new spinal cord relapse with worsening lower limb weakness and a new sensory level at T10. He was admitted to the hospital and treated with intravenous methylprednisolone 1 g for five days. Subsequently, he was placed on intravenous methylprednisolone 1 g weekly for six weeks, followed by high-dose maintenance of 60 mg oral prednisolone. The patient’s weakness eventually improved. However, frequent flare-ups were observed with steroid taper before the initiation of steroid-sparing agents. Intravenous immunoglobulin (IVIG) was tried during the course, which elicited no clinical response. The patient was started on azathioprine but had abnormal liver function tests and was, hence, weaned off the medication. Rituximab (RTX) infusion protocol was initiated. The patient received two doses of 1 g, spaced in time with an interval of two weeks. This was followed by repeated cycles every six months. The patient showed significant clinical improvement after receiving RTX, and we were able to taper down the dose of prednisone up to 10 mg. After one-year follow-up from starting RTX, his motor examination showed mild persistent weakness in the left ankle dorsiflexion graded as 4 out 5, but otherwise normal strength throughout. His current EDSS is 2.5.

## Discussion

CCPD is a rare entity with no well-established diagnostic criteria. To our knowledge, no case was reported in Saudi Arabia of a patient with CCPD. A retrospective cohort study of 31 patients was conducted in two centers in Italy [1]. The study aimed to identify clinical features, diagnostic findings, and possible treatments of CCPD. The majority of patients were men with a disease onset at 57 years of age. A total of 20 participants had an infection or received a vaccination before their presentation. Spinal cord lesions were anticipated as many of the enrolled patients suffered from lower-limb sensory-motor impairment and sphincter dysfunction. Nevertheless, altered mental status and cranial nerve involvement can be the presenting symptoms of CCPD patients. The disease course in the study’s participants was monophasic in one-third of the cases, which indicates a single isolated episode of demyelination. A total of 21 patients showed progression of the disease, either by relapse with subacute onset of new symptoms or by a constant chronic progression. Interestingly, six cases with distal paresthesia presented with a progressive disease course from the onset of symptoms. Most patients in the acute phase were treated with steroids, IVIG, or plasma exchange therapy. A total of 19 out of 26 patients had an improvement in the modified Rankin Scale (mRS) score by at least one point. Twenty-four patients with relapses or chronic progression had a poorer response rate to steroids or IVIG when used later during the disease course.

Neurofascin is a member of the L1 subgroup of adhesion molecules expressed at the nodes of Ranvier and the paranodes in both the CNS and PNS [5]. In a Japanese study, Kawamura et al. discovered that anti-neurofascin (anti-NF) antibody was present in 86% of CCPD patients [4]. The Japanese study also displayed a better response to IVIG or plasma exchange treatment in patients with positive anti-NF antibodies, addressing the significance of its detection. However, our patient tested negative for anti-NF antibody. This is in alignment with a previous study done on Caucasians to assess the presence of the same antibody among CCPD patients [6]. In that study, none of the patients with CCPD were found to be positive for the anti-NF antibody. Nevertheless, the study speculated that these findings were partially attributable to the participants’ ethnicity and the heterogeneity of their clinical presentation.

In our patient, RTX was successfully used to taper down the dose of steroids. The use of RTX has been reported in several studies. In a placebo-controlled randomized controlled trial of 104 relapsing-remitting MS (RRMS) patients, RTX was found to drastically decrease the counts of contrast-enhancing lesions as well as volumes of T2 lesions. Furthermore, it reduced the proportion of patients with relapse at 48 weeks (RTX: 20.3% vs. placebo: 40.0%) [7]. A population-based Swedish study of 494 patients with newly diagnosed RRMS concluded that the use of RTX decreases the annual relapse rate as well as neuroradiologic disease activity in comparison with all other disease-modifying therapies [8]. RTX was used in a case series of 11 patients with CIDP that showed evidence of improvement. The Medical Research Council sum score showed a change ranging from 0 to 60 post-treatment. Additionally, the change in Inflammatory Neuropathy Cause and Treatment disability score ranged from 0 to 8 after starting the treatment with a mean of 4.54 [9]. A case report of a 14-year-old patient with CCPD, who was treated with RTX, revealed both a clinical improvement in sensory and motor deficit as well as a reduction in the number of lesions on brain and spine MRI [10].

## Conclusions

RTX yielded excellent clinical improvement in our case, signifying a potential efficacy of B-cell depleting therapy in CCPD. This study reports a rare neurological disease entity and highlights the necessity for conducting further studies to optimally demonstrate the efficacy of RTX in CCPD.
